# Potential Ketoacidosis Linked to Imeglimin and Metformin Co-administration in a Patient With Type 2 Diabetes

**DOI:** 10.7759/cureus.40702

**Published:** 2023-06-20

**Authors:** Hirofumi Yamagishi, Sachina Hoshino, Akiko Hirano, Atsushi Oshima, Taihei Imai

**Affiliations:** 1 Department of Endocrinology and Metabolism, JA Toride General Medical Center, Toride City, JPN

**Keywords:** ketoacidosis, drug interaction, combination therapy, metformin, imeglimin

## Abstract

A 74-year-old woman with type 2 diabetes mellitus developed ketoacidosis within six days of adding metformin to imeglimin treatment. The patient was insulin-sensitive and showed preserved insulin secretion; therefore, insulin insufficiency alone was unlikely to contribute to the development of ketoacidosis. Both imeglimin and metformin partially inhibit complex I in the mitochondrial respiratory chain. Inhibition of mitochondrial respiration can lead to tricarboxylic acid (TCA) cycle suppression. Thus, the entry of acetyl-coenzyme A into TCA cycle is restricted, and it is eventually used in ketogenesis. Therefore, the combination of imeglimin and metformin might have precipitated the development of ketoacidosis.

## Introduction

Imeglimin, a first-in-class drug for treating type 2 diabetes mellitus, was approved in June 2021 as a novel oral antihyperglycemic agent by the Pharmaceuticals and Medical Devices Agency in Japan. Imeglimin is synthesized from metformin in a single chemical reaction [1]. Both imeglimin and metformin partially inhibit complex I in the mitochondrial respiratory chain and share some modes of action, including the inhibition of hepatic glucose output [2,3]. However, unlike metformin, imeglimin amplifies glucose-stimulated insulin secretion [4]. Therefore, imeglimin is administered in combination with metformin as an add-on treatment. Several studies have shown favorable safety and tolerability of combination therapy with imeglimin and metformin [5,6]. However, the sample sizes in these studies were too small to detect rare complications. Therefore, we reported an atypical case of ketoacidosis in a diabetic patient possibly associated with the combination of imeglimin and metformin.

## Case presentation

The patient was a 74-year-old woman diagnosed with diabetic ketoacidosis (DKA). Her past medical history included type 2 diabetes mellitus and dyslipidemia. There was no history of hypertension, chronic kidney disease, or psychiatric disorders. The patient was prescribed dulaglutide (0.75 mg weekly), imeglimin (1,000 mg twice daily), repaglinide (0.5 mg thrice daily), and miglitol (75 mg thrice daily) for type 2 diabetes mellitus, along with pitavastatin (1 mg once daily) for dyslipidemia. The patient denied the use of alcohol and illicit drugs. Despite the suboptimal body weight, she prepared her own meals and maintained regular dietary habits. Metformin (250 mg twice daily) was administered six days before presentation. At the time, urinalysis did not reveal ketonuria. The patient experienced nausea promptly upon initiating metformin therapy.

She visited the outpatient department with chief complaints of abdominal pain and vomiting. Her body mass index was 18.2 kg/m^2^ (height of 151 cm and body weight of 41.4 kg). Furthermore, a physical examination revealed a mild disturbance of consciousness, mucosal dryness, and tenderness in the upper abdomen. No fever or respiratory symptoms were observed. Laboratory tests revealed hyperglycemia (blood glucose level of 245 mg/dL) with acidemia (pH, 7.30), low serum bicarbonate level (14.8 mmol/L), and ketonemia (total ketone level of 8.0 mmol/L) (Table 1).

**Table 1:** Laboratory findings 3-HBA, 3-hydroxybutyric acid; AA, acetoacetate; AG, anion gap; ALP, alkaline phosphatase; ALT, alanine transaminase; AMY, amylase; AST, aspartate aminotransferase; Bil, bilirubin; BUN, blood urea nitrogen; CK, creatine kinase; Cl, chloride; Cre, creatinine; CRP, C-reactive protein; GAD Ab, anti-glutamic acid decarboxylase antibody; γ-GTP, γ-glutamyl transferase; Glu, glucose; Hb, hemoglobin; HbA1c, hemoglobin A1c; HCO_3_, bicarbonate; IA-2 Ab, anti-islet antigen 2 antibody; IGF-1, insulin-like growth factor 1; K, potassium; LDH, lactate dehydrogenase; M, metanephrine; NM, normetanephrine; Na, sodium; Plt, platelets; T3, triiodothyronine; T4, thyroxine; TSH, thyroid stimulating hormone; WBC, white blood cells

Laboratory test	Results	Reference range
pH	7.300	7.35-7.45
HCO₃ (mmol/L)	14.8	21-28 mmol/L
AG (mmol/L)	23.2	10-20 mmol/L
lactate (mmol/L)	2.9	0.5-1.6 mmol/L
Ketones in urine	(4+)	(–)
Leukocytes in urine	(–)	(–)
Nitrites in urine	(–)	(–)
WBC (×1,000 /µL)	5.71	3.3-8.6 ×1,000 /µL
Hb (g/dL)	15.6	11.6-14.8 g/dL
Plt (×10,000 /µL)	19.4	15.8-34.8 ×10,000 /µL
BUN (mg/dL)	40	8-20 mg/dL
Cre (mg/dL)	0.72	0.46-0.79 mg/dL
Na (mEq/L)	133	138-145 mEq/L
K (mEq/L)	4.5	3.6-4.8 mEq/L
Cl (mEq/L)	100	101-108 mEq/L
AST (U/L)	20	13-30 U/L
ALT (U/L)	20	7-23 U/L
LDH (U/L)	182	124-222 U/L
ALP (U/L)	32	38-113 U/L
γ-GTP (U/L)	19	9-32 U/L
Total Bil (mg/dL)	2.87	0.40-1.50 mg/dL
AMY (U/L)	24	44-132 U/L
CK (U/L)	72	41-153 U/L
CRP (mg/dL)	<0.10	0.00-0.14 mg/dL
Glu (mg/dL)	245	73-109 mg/dL
HbA1c (%)	9.4	4.9-6.0%
Total ketone (mmol/L)	8.0	<0.13 mmol/L
3-HBA (mmol/L)	6.0	<0.085 mmol/L
AA (mmol/L)	2.0	<0.055 mmol/L
GAD Ab (U/mL)	<5.0	Negative
IA-2 Ab (U/mL)	<0.6	Negative
Urinary C-peptide (μg/day)	62.7	29.2-167 μg/day
Serum C-peptide (ng/dL)	1.08	0.61-2.09 ng/dL
TSH (μIU/mL)	0.895	0.35-4.94 μIU/mL
Free T4 (ng/dL)	0.84	0.70-1.48 ng/dL
Free T3 (pg/mL)	1.89	1.68-3.67 pg/mL
M (pg/mL)	54	<130 pg/mL
NM (pg/mL)	<30	<506 pg/mL
IGF-1 (ng/mL)	128	53-165 pg/mL

Based on these findings, the patient was diagnosed with DKA [7,8]. As hepatobiliary enzymes and amylase were not elevated and abdominal computed tomography (CT) revealed no significant findings, the presence of concomitant acute abdomen such as acute pancreatitis, cholecystitis, or cholangitis was ruled out. Moreover, given that the white blood cell count and C-reactive protein were within the normal range, the absence of pyuria was noted, and no remarkable findings were made on chest CT, the likelihood of infectious complication, such as urinary tract infection or pneumonia, was not considered. The electrocardiogram showed no ST-segment elevation, and myocardial infarction was not suspected.

The clinical management included intravenous insulin administration, fluid restoration, and potassium infusion. As blood ketone and glucose levels decreased rapidly after treatment initiation, the dose of intravenous insulin was reduced to 0.2 units/hour and switched to subcutaneous insulin on the day of hospitalization.

Interestingly, ketoacidosis developed within six days, suggesting fulminant type 1 diabetes mellitus [9]. However, fasting serum C-peptide and 24-hour urinary C-peptide levels showed preserved insulin secretion (Table 1). Insulin therapy was discontinued on day 10 of hospitalization, and the patient was discharged on day 14. The prescription at discharge was dulaglutide (0.75 mg weekly), metformin (250 mg twice daily), glimepiride (0.5 mg once daily), and pitavastatin (1 mg once daily). Neither nausea nor ketonuria was observed during the outpatient clinic visit following discharge.

## Discussion

This was an atypical case of DKA associated with the combined use of imeglimin and metformin. First, the patient rapidly developed ketoacidosis with preserved insulin secretion. Despite poor glycemic control, the patient was sensitive to insulin. Therefore, insulin insufficiency alone unlikely caused the development of ketoacidosis. Second, blood glucose levels were relatively low despite the substantial elevation in blood ketones levels. Therefore, predisposing factors in addition to insulin insufficiency might have promoted ketogenesis.

Immediately after initiation of metformin, the patient experienced nausea and was unable to eat sufficiently. Because the combined use of biguanide and imeglimin increases gastrointestinal symptoms such as diarrhea compared to imeglimin monotherapy [5], the occurrence of nausea may be linked to the simultaneous coadministration of metformin and imeglimin. The inability to eat due to nausea likely contributed to ketogenesis. However, while mild ketosis generally develops after 12 to 14 hours of fasting, starvation ought to persist for a minimum duration of 20-30 days before the blood ketone concentrations rise to 8-10 mmol/L [10,11]. Hence, the occurrence of nausea, which began six days prior to the presentation, does not adequately account for the etiology of ketoacidosis.

In adults with known diabetes mellitus, precipitating factors for DKA include infections, intercurrent illnesses such as coronary syndrome, trauma, and alcohol abuse [8,12]. In our patients, there were no complications of infection or acute illness such as myocardial infarction or acute pancreatitis. Various medications that alter carbohydrate metabolism, such as corticosteroids, diuretics, pentamidine, sympathomimetic agents, atypical antipsychotic agents, and immune checkpoint inhibitors may also precipitate the development of DKA [8,12]. The use of sodium-glucose cotransporter 2 (SGLT-2) inhibitors has also been implicated in the development of euglycemic DKA [12,13]. A comprehensive medication review revealed that our patient was not taking any of the aforementioned medications. As described above, no known precipitating factors for DKA were found in our patient. Therefore, we hypothesized that the combination of imeglimin and metformin may have contributed to the development of ketoacidosis.

In DKA, insulin deficiency combined with increased levels of catecholamine, cortisol, and growth hormone triggers the activation of hormone-sensitive lipase, thereby initiating the breakdown of triglycerides into fatty acids [12,14]. Subsequently, the liberated fatty acids undergo oxidation to form acetyl coenzyme A (CoA), which enters the tricarboxylic acid (TCA) cycle to generate cellular energy. However, when the production of acetyl CoA exceeds the oxidative capacity of the TCA cycle, it is diverted toward ketogenesis [12,15]. Hence, the diminished oxidative capacity of the TCA cycle can potentially contribute to the occurrence of ketonemia. Indeed, this factor contributes to the development of alcoholic ketoacidosis (AKA), which follows a distinct pathogenesis compared to DKA [16,17]. In AKA, ethanol oxidation involves the reduction of nicotinamide adenine dinucleotide (NAD) to dihydronicotinamide adenine dinucleotide (NADH). The increased NADH/NAD ratio inhibits TCA cycle, thereby promoting ketogenesis [16,17].

Regarding our patient, she developed ketoacidosis within six days after adding metformin to imeglimin treatment. Although both imeglimin and metformin partially inhibit complex I in the mitochondrial respiration chain, their modes of inhibition are different [2]; imeglimin is a competitive inhibitor, whereas metformin is a noncompetitive inhibitor. The inhibition of mitochondrial respiration can lead to the suppression of the TCA cycle. Thus, the entry of acetyl-CoA into the TCA cycle is restricted, and it is eventually used in ketogenesis. Therefore, the combination of the two drugs can induce ketogenesis under certain conditions.

This study highlights a potential safety concern with the concurrent use of imeglimin and metformin. Healthcare providers should be aware of the risk and consider careful monitoring when initiating the combination for treating diabetic patients. Due to the limited scope of a single case report, further research is required to establish a definitive association between the combined use of imeglimin and metformin and the development of ketoacidosis.

## Conclusions

This report described an atypical case of DKA that developed immediately after the administration of metformin in combination with imeglimin. Imeglimin and metformin share similar pharmacological effect through different modes of action. However, the interaction between imeglimin and metformin remains unknown. Therefore, further accumulation of cases is required in the future.
